# Supplementary material for: Effects of a simulated marine heatwave on the structure and composition of Mediterranean plankton in a mesocosm study
Source: PLoS One. 2025 Nov 21;20(11):e0337112. doi: 10.1371/journal.pone.0337112 (PMC12637984; doi:10.1371/journal.pone.0337112)
Supplement: S1 Table — All species identified across the different planktonic groups are listed to highlight overall richness. Some species were not discussed in the main text due to their very low abundance. Autotrophic (A); Mixotrophic (M); Heterotrophic (H); Undetermined (U). (DOCX) [file pone.0337112.s001.docx]

**S1 Table.** **Summary of identified organisms using microscopy and feeding strategies derived by literature.**

| **Parameter** | | **Methods of analysis** | **Regime attribution** | **References** |
| --- | --- | --- | --- | --- |
| **Abiotic** | Temperature | Temperature probes | x |  |
|  | Salinity | High frequency probes | x |  |
|  | Light |  | x |  |
| **Nutrient** | Si | Automated colorimeter | x |  |
|  | PO_4_^3-^ |  | x |  |
|  | N |  | x |  |
|  | Chlorophyll-a | HPLC | A |  |
|  | Viruses | Flow cytometry | H |  |
|  | Bacteria |  | H |  |
| **Small phytoplankton < 10 µm** | Cyanobacteria |  | A |  |
|  | Picophytoplankton eukaryotes |  | A |  |
|  | Nanophytoplankton eukaryotes |  | A |  |
| **Diatoms <5 µm** | *Bacteriastrum parallelum* | Inverted microscope | A | Sarno D.,Zingone, A& Marino, D., 1997 |
|  | *Chaetoceros* spp. |  | A | Ehrenberg, C.G (1844) |
|  | *Cyclotella* spp. |  | A | Berisson, [L.] A. (1838) |
|  | *Cylindrotheca closterium* |  | A | Reimann, N.E.F. & Lewin, J.C., (1964) |
|  | Pennate diatoms inf 10 |  | A |  |
|  | Pennate diatoms sup 10 |  | A |  |
| **Flagellates <5 µm** | *Dictyocha speculum* | Inverted microscope | A | Ehrenberg, C.G (1839) |
|  | *Pseudoscourfieldia marina* |  | A | Throndsen (1969) |
|  | Cryptophyceae inf 10 |  | A and M | Paul C.Silva (1980) & Taylor, William D (1988) |
|  | Cryptophyceae sup 10 |  | A and M | T.Mackiewicz (1991) |
|  | Euglenophyceae |  | A, M, and H | T. Cavalier-Smith (2016) |
|  | *Dinobryon faculiferum* |  | M | Willen 1992 |
|  | *Ollicola vangoorii* |  | M | Novarino, Oliva, and Pérez-Uz (2002) |
|  | *Leucocryptos marina* |  | H | Butcher 1967, Vørs, Naja. (1992) |
| **Dinoflagellates <5 µm** | *Heterocapsa niei* | Inverted microscope | A | L.C.Morrill & A.R.Loeblich (1981) |
|  | *Prorocentrum triestinum* |  | A | Schiller (1918) |
|  | Naked dinoflagellate inf 15 |  | U |  |
|  | Naked dinoflagellates sup 15 |  | U |  |
|  | Thecate dinoflagellate inf 15 |  | U |  |
|  | Thecate dinoflagellate sup 15 |  | U |  |
|  | Calciodinelloideae |  | U |  |
|  | *Alexandrium* spp. |  | M | Halim (1960), Anderson et al. (2012) |
|  | *Prorocentrum gracile* |  | M | Schutt, F.(1895) |
|  | *Gyrodinium* spp. |  | H | Atwood Kofoid, and Swezy (1921) |
|  | *Protoperidinium bipes* |  | H | Balech (1974) |
|  | *Protoperidinium diabolus* |  | H | Balech (1974) |
| **Ciliates** | Aloricates | Inverted microscope | H |  |
|  | Tintinnids |  | H |  |

All species identified across the different planktonic groups are listed to highlight overall richness. Some species were not discussed in the main text due to their very low abundance. Autotrophic (A); Mixotrophic (M); Heterotrophic (H); Undetermined (U).
