# Supplementary material for: Effects of a simulated marine heatwave on the structure and composition of Mediterranean plankton in a mesocosm study
Source: PLoS One. 2025 Nov 21;20(11):e0337112. doi: 10.1371/journal.pone.0337112 (PMC12637984; doi:10.1371/journal.pone.0337112)
Supplement: S2 Table — (a) Kruskal-Wallis test, (b) repeated-measures ANOVA. Only the variables with at least one significant p-value (≤ 0.05) across the three periods are shown in bold, indicating a significant difference between the two conditions. (DOCX) [file pone.0337112.s002.docx]

**S2 Table**. **P-value results, comparing conditions in plankton community species, genus, and group.**

(a) Kruskal-Wallis test, (b) repeated-measures ANOVA.

Only the variables with at least one significant p-value (≤0.05) across the three periods are shown in bold, indicating a significant difference between the two conditions.

| Parameters | Periods | | | | | | | | |
| --- | --- | --- | --- | --- | --- | --- | --- | --- | --- |
|  | **HW (d2-10)** | | | **PostHW (d11-20)** | | | **ALL (d2-20)** | | |
|  | df | Test value | *p-value* | df | Test value | *p-value* | df | Test value | *p-value* |
| Cylindrotheca closterium | 1 | 0.37 | 0.544 (a) | 1 | 0.09 | 0.769 (a) | 1 | 0.02 | 0.885 (a) |
| Bacteriastrum parallelum | 1 | 0.08 | 0.781 (a) | 1 | 3.69 | 0.055 (a) | 1 | 1.58 | 0.208 (a) |
| Chaetoceros sp. | 19 | 0.27 | 0.609 (b) | 1 | 1.25 | 0.264 (a) | 1 | 1.06 | 0.304 (a) |
| Pennate diatoms > 10 µm | 1 | 0.47 | 0.494 (a) | 19 | 1.68 | 0.210 (b) | 1 | 1E^-4^ | 0.991 (a) |
| Pennate diatoms < 10 µm | **1** | **7.50** | **0.006 (a)** | 1 | 2.52 | 0.113 (a) | **1** | **9.25** | **0.002 (a)** |
| Cyclotella spp. | **19** | **10.21** | **0.005** **(b)** | 1 | 0.64 | 0.425 (a) | **1** | **5.40** | **0.020 (a)** |
| Pseudiscoufielda marina | **1** | **4.61** | **0.032 (a)** | 1 | 0.8E^--4^ | 0.976 (a) | 1 | 2.23 | 0.135 (a) |
| Prorocentrum triestinum | 1 | 0 | 1 (a) | 1 | 2.28 | 0.131 (a) | 1 | 1.05 | 0.305 (a) |
| Ollicola vangoorii | **19** | **17.14** | **<0.001 (b)** | **21** | **4.57** | **0.045 (b)** | **41** | **19.01** | **<0.001 (b)** |
| Dinobryon faculiferum | 19 | 0.01 | 0.923 (b) | 1 | 0.65 | 0.418 (a) | 1 | 0.15 | 0.699 (a) |
| Naked dinoflagellates < 15 µm | **19** | **8.47** | **0.009 (b)** | **19** | **3.51** | **0.077 (b)** | **39** | **11.64** | **0.004 (b)** |
| Thecate dinoflagellates < 15 µm | 1 | 3.33 | 0.682 (a) | **1** | **5.97** | **0.015 (a)** | **1** | **9.35** | **0.002 (a)** |
| Prorocentrum gracile | 1 | 0.66 | 1 (a) | 1 | 0.78 | 0.782 (a) | 1 | 0.15 | 0.701 (a) |
| HNF < 3 µm | 19 | 1.08 | 0.311 (b) | **24** | **6.11** | **0.021 (b)** | 44 | 1.30 | 0.260 (b) |
| HNF 3-5 µm | **19** | **25.53** | **<0.001 (b)** | **24** | **5.63** | **0.026 (b)** | **44** | **24.51** | **<0.001 (b)** |
| HNF > 5 µm | 19 | 0.55 | 0.469 (b) | 1 | 0.11 | 0.739 (a) | 44 | 0.14 | 0.710 (b) |
