# Supplementary figures and images for: Effects of a simulated marine heatwave on the structure and composition of Mediterranean plankton in a mesocosm study
Source: PLoS One. 2025 Nov 21;20(11):e0337112. doi: 10.1371/journal.pone.0337112 (PMC12637984; doi:10.1371/journal.pone.0337112)

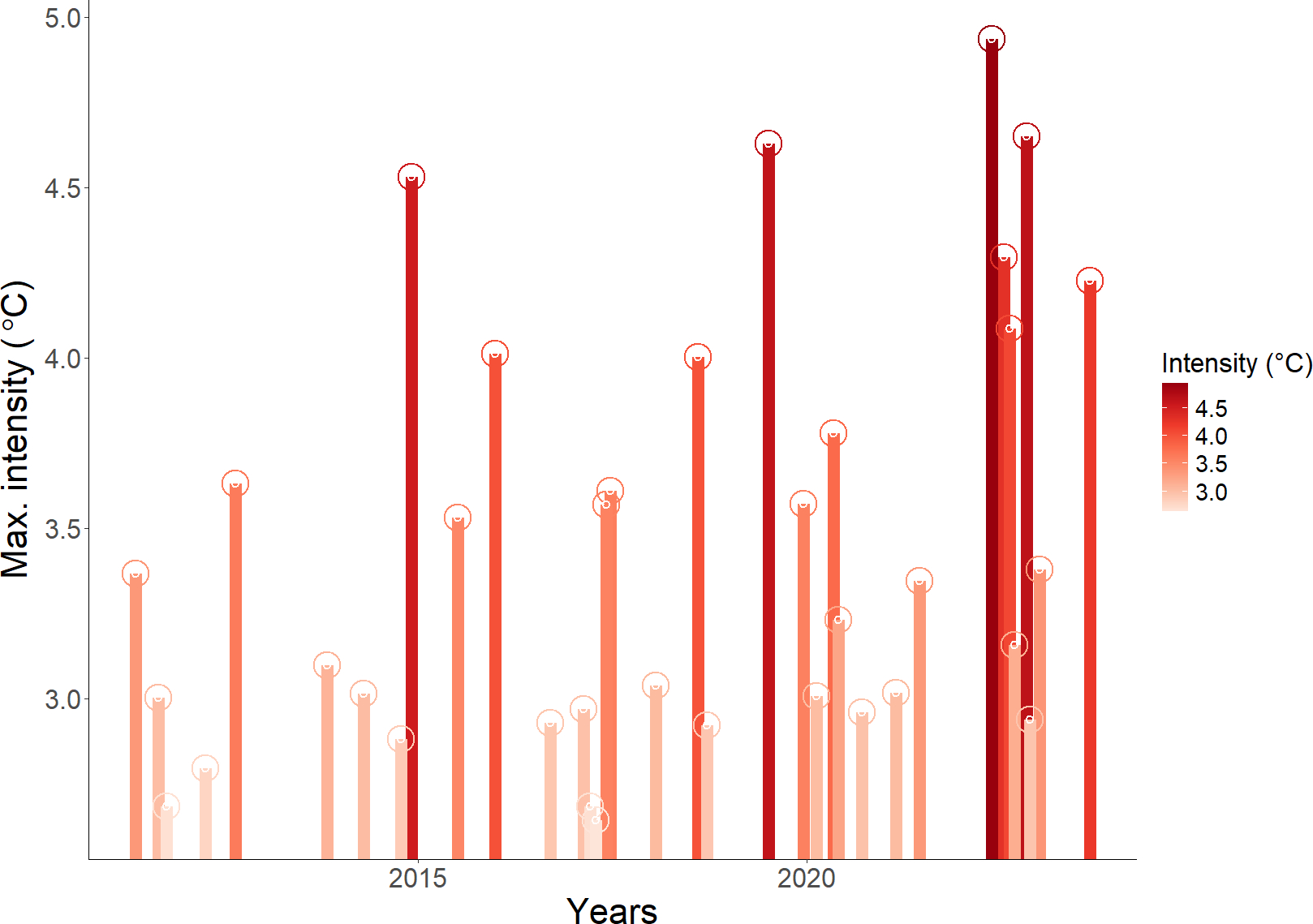

Supplement: S1 Fig — The data coming from ECOSCOPA network from 2011 to 2023. (TIF) [file pone.0337112.s003.tif]

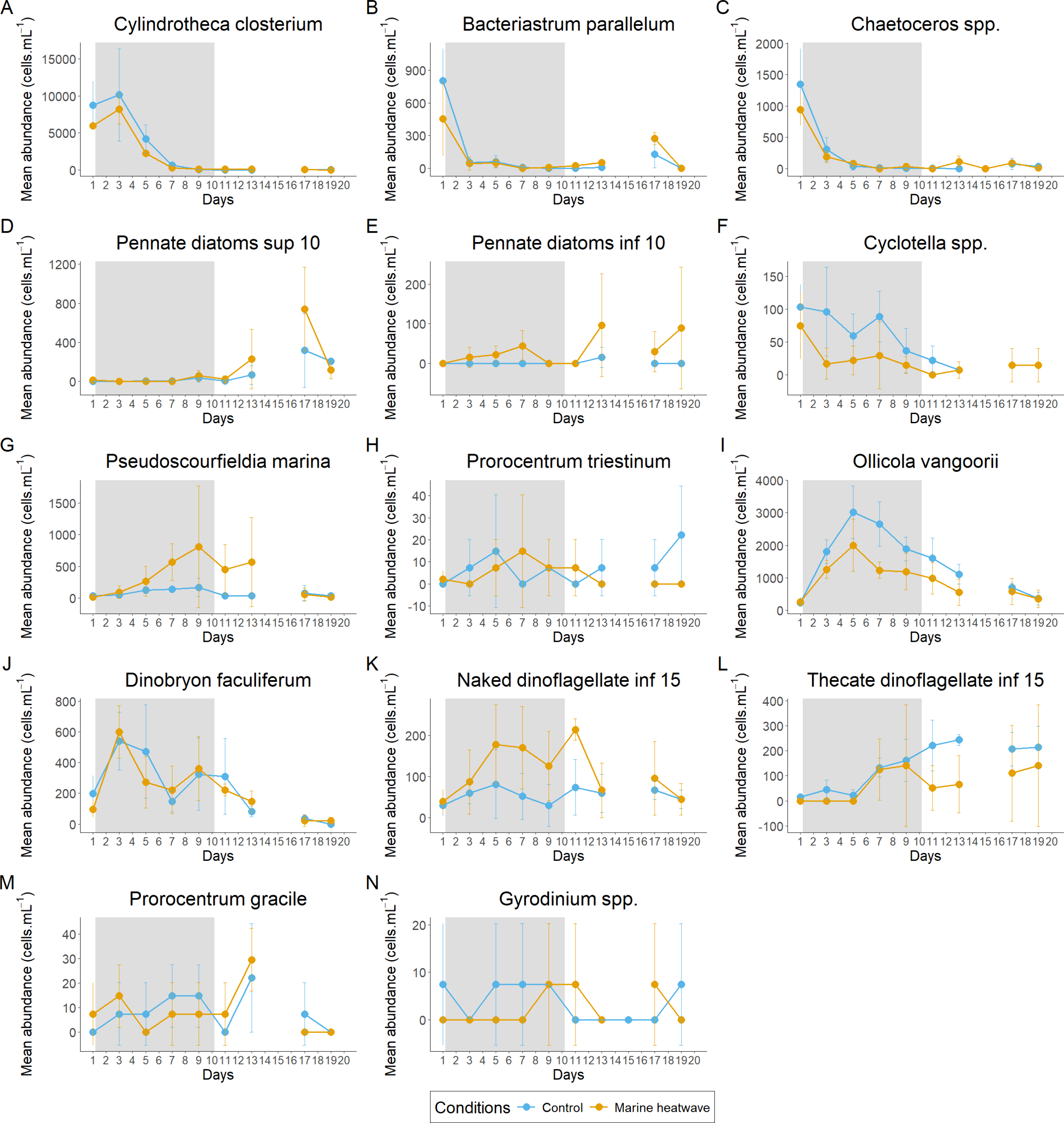

Supplement: S2 Fig — Diatoms communities: (A) Cylindrotheca closterium, (B) Bacteriastrum parallelum, (C) Chaetoceros spp, (D) the pennate diatoms with the size superior to 10 µm (pennate > 10 µm), and (E) with a size under 10 µm (pennate < 10 µm), (F) Cyclotella spp., autotrophic flagellates: (G) Pseudoscourfielda marina, autotrophic dinoflagellates; (H) Prorocentrum triestinum, mixotrophic flagellates: (I) Ollicola vangoorii and (J) Dinobryon faculciferum, undetermined dinoflagellates: (K) naked dinoflagellates < 15, (L) thecate dinoflagellates < 15, mixotrophic dinoflagellates: (M) Prorocentrum gracile, heterotrophic dinoflagellates: (N) Gyrodinium spp. Orange and blue circles correspond to heated and control conditions, respectively. The gray area corresponds to the + 3 °C heating phase. Error bars indicate standard deviation of the mean. (TIF) [file pone.0337112.s004.tif]

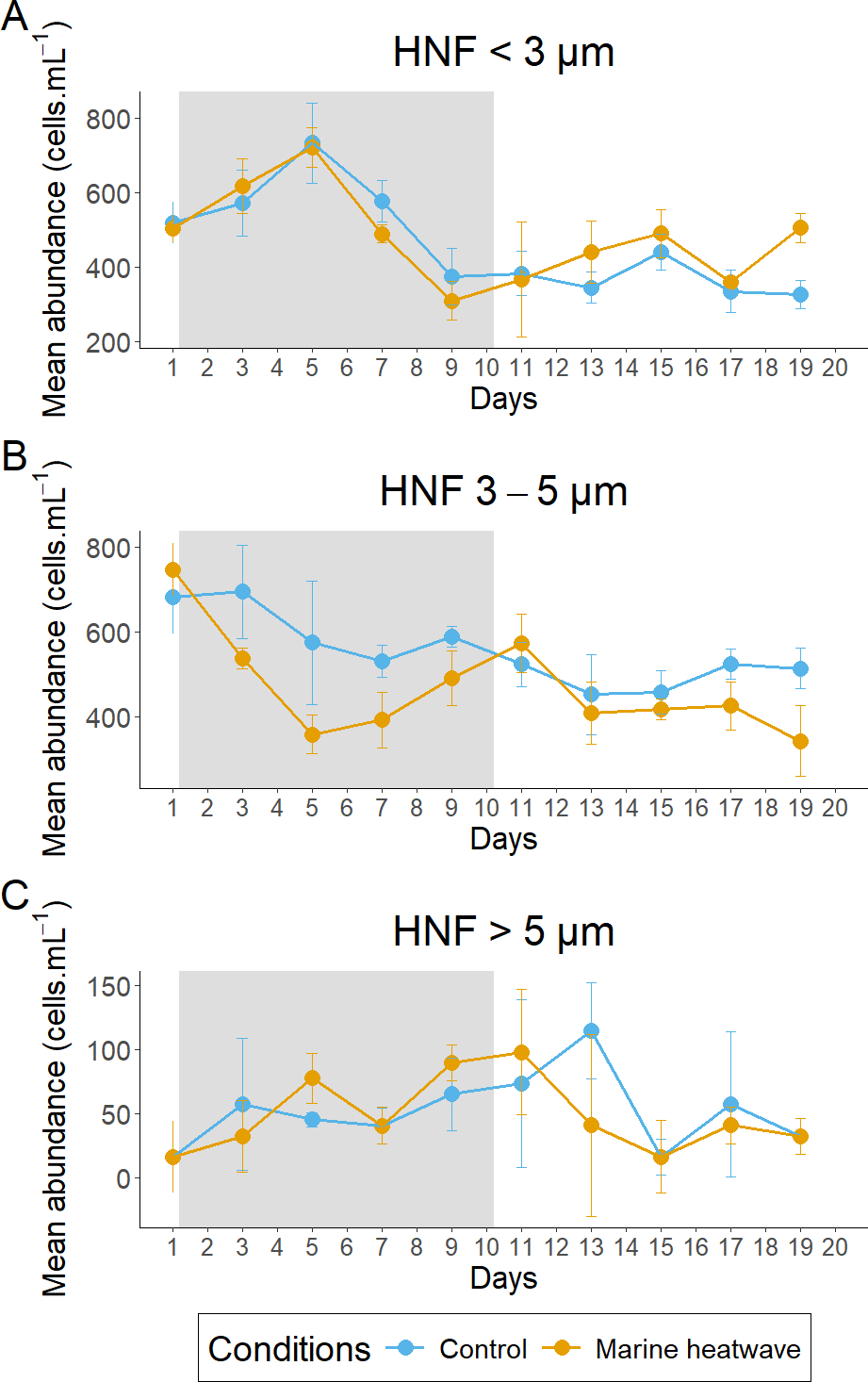

Supplement: S3 Fig — (A) with body size inferior to 3 µm, HNF < 3 µm, (B) between 3 µm and 5 µm, HNF 3–5 µm, and (C) superior to 5 µm, HNF > 5 µm. Orange and blue circles correspond to heated and control conditions, respectively. The gray area represents the marine heatwave period (d2-d10). Error bars indicate standard deviation of the mean. (TIF) [file pone.0337112.s005.tif]
