# Supplementary material for: Effects of a simulated marine heatwave on the structure and composition of Mediterranean plankton in a mesocosm study
Source: PLoS One. 2025 Nov 21;20(11):e0337112. doi: 10.1371/journal.pone.0337112 (PMC12637984; doi:10.1371/journal.pone.0337112)
Supplement: Attachment — Submitted filename: Responses to Reviewers.docx [file pone.0337112.s007.docx]

Manuscript ID: **PONE-D-25-36140**

Title: **Effects of a simulated marine heatwave on the structure and composition of Mediterranean plankton in a mesocosm study.**

Dear Editor, Dear Reviewers,

We sincerely thank you for the time you have devoted to our manuscript and for your valuable comments. We have carefully considered all your remarks and revised the article accordingly. Below you will find a detailed response to each of the points raised.

You will find below a description of the layout changes made to highlight the changes made to the manuscript following comments.

- Changes and additions to the text are highlighted in yellow. Example: Adding text
- The parts or elements removed from the manuscript are crossed out and colored red. Example: ~~text deletion~~
- Movement of sentences or paragraphs to another location in the revised version are indicated in green. Example: text movement

The numbers of lines mentioned below correspond to those of the “Revised Version Without Track Changes”.

**Journal Requirements:**

**Requirement 1:**

Please ensure that your manuscript meets PLOS ONE's style requirements, including those for file naming.

**Response 1:**

The manuscript was revised and supplementary files to comply with PLOS ONE’s style and file-naming requirements.

**Requirement 2:**

We note that the grant information you provided in the ‘Funding Information’ and ‘Financial Disclosure’ sections do not match.

When you resubmit, please ensure that you provide the correct grant numbers for the awards you received for your study in the ‘Funding Information’ section.

**Response 2:**

The grant information was corrected so that it is consistent between the ‘Funding Information’ and ‘Financial Disclosure’ sections, and the correct grant numbers are now provided.

**Requirement 3:**

When completing the data availability statement of the submission form, you indicated that you will make your data available on acceptance. We strongly recommend all authors decide on a data sharing plan before acceptance, as the process can be lengthy and hold up publication timelines. Please note that, though access restrictions are acceptable now, your entire data will need to be made freely accessible if your manuscript is accepted for publication. This policy applies to all data except where public deposition would breach compliance with the protocol approved by your research ethics board. If you are unable to adhere to our open data policy, please kindly revise your statement to explain your reasoning and we will seek the editor's input on an exemption. Please be assured that, once you have provided your new statement, the assessment of your exemption will not hold up the peer review process.

**Response 3:**

The Data Availability Statement was updated and data underlying this study was already published in SEANOE (https://doi.org/10.17882/108308) and are now fully accessible, in accordance with PLOS ONE’s open data policy. DOI of published data was also mentioned in the Revised Version in Lines 215-216.

**Requirement 4:**

Please amend either the title on the online submission form (via Edit Submission) or the title in the manuscript so that they are identical.

**Response 4:**

The title was amended to ensure that it is identical in both the manuscript and the online submission form.

**Requirement 5:**

If the reviewer comments include a recommendation to cite specific previously published works, please review and evaluate these publications to determine whether they are relevant and should be cited. There is no requirement to cite these works unless the editor has indicated otherwise.

**Response 5:**

As there was no specific recommendation for additional citation was asked by the Reviewers, any change to the reference list was made.

**Requirement 6:**

Please review your reference list to ensure that it is complete and correct. If you have cited papers that have been retracted, please include the rationale for doing so in the manuscript text or remove these references and replace them with relevant current references. Any changes to the reference list should be mentioned in the rebuttal letter that accompanies your revised manuscript. If you need to cite a retracted article, indicate the article’s retracted status in the References list and also include a citation and full reference for the retraction notice.

**Response 6:**

We have carefully checked and revised the reference list to ensure completeness and accuracy. No retracted articles remain cited.

**Reviewers’ comments:**

**Reviewer #1 – comment 1:**

“Line 143, you already mentioned shortening. Thus, you should not multiply it, as in the line 160 and others (l 262, table later…. Maybe better to live in a line 160 and delete an explanation of the shortening before.”

**Reviewer #1 – response to comment 1:**

Thank you for this suggestion. We agree with this comment and remove the explanation of the abbreviation at lines 143, 262, and 281, (lines 143, 265, and 284 in revised version) keeping it only at line 160 as recommended. This modification avoids redundancy and improves the clarity of the manuscript.

**Reviewer #1 – comment 2:**

“Line 274, please correct ‘-‘ sign for NO2 and NO3, it should be upper.”

**Reviewer #1 – response to comment 2:**

Thanks for this comment. The notations of NO_2_ and NO_3_ were corrected by placing the minus sign (“-”) at the upper position (NO_2_^-^ and NO_3_^-^ ) in line 277.

**Reviewer #1 – comment 3:**

“Line 291, 294, 296, 297, 315, 316 etc for cells.ml, ‘.’ Should be in the middle of it to differentiate between dot and the multiply sign.

Also, I noticed that sometimes you have Litters (L) and sometimes ml. It should be unified according to CI.”

**Reviewer #1 – response to comment 3:**

Thank you for this remark. As suggested, we have replaced the dot (“.”) with the middle dot (“·”) in all relevant expressions, so that “cells.ml^-1^” now appears as “cells·mL^-1^” (lines 294, 297-301, 314, 318, 319, 321, 325-327, 356, 360, 376, 377, and 380).

Following the SI unit’s brochure, we have throughout the manuscript also

- Standardized volume units to “mL” (instead of “L” or “ml”),
- Harmonized the notation “cells·mL^-1^”, and
- Added appropriate spacing between numbers and units/symbols (e.g., °C, %, <, >, and =).

**Reviewer #1 – comment 4:**

“In line 247 you explained the shortening of PCA, please do it only once, there. It is not necessary to repeat as for example in line 415.”

**Reviewer #1 – response to comment 4:**

As suggested, the repeated explanation was removed at line 418 and retained the definition only at line 250 to avoid redundancy.

**Reviewer #1 – comment 5:**

“Line 614 species is repeated.”

**Reviewer #1 – response to comment 5:**

The redundancy was corrected by removing the repeated word *species* at line 634.

**Reviewer #1 – comment 6:**

“Please add to Line 586, what other groups you mean.”

**Reviewer #1 – response to comment 6:**

Thanks for this suggestion. To clarify this, the groups referred to “other groups” were specified in lines 586. The revised sentence now reads:

Line 605. *“Cyanobacteria, picophytoplankton, diatom, autotrophic, and mixotrophic flagellates showed the highest positive resilience slopes compared to the other groups (viruses, bacteria, nanophyto, HNF, all trophic modes dinoflagellates, and ciliates) that exhibited negative or near-zero slopes.”*

**Reviewer #2 – comment 1:**

“I think it could benefit that incluid from metazooplankton data: **Although mentioned in the discussion, including more details on their dynamics and relationship with protozooplankton could enrich the analysis, as well as the impact on biogeochemical processes: Explore how changes in plankton communities affect processes such as the carbon cycle or oxygen production**.”

**Reviewer #2 – response to comment 1:**

We fully agree with this point. Accordingly, we expanded the discussion by including more information on mesozooplankton dynamic, their interactions within food web, and their influence on biogeochemical cycles. Two new paragraphs therefore were added to the Revised Version:

- Lines 504 – 511:

*“Additionally, Zervouldaki et al. (2024) reported that the copepods, polychaetes, larvae of bivalve, and gastropods, were the dominant mesozooplankton groups present in the mesocosms during the same experiment.* *The MHW condition had a significant positive effect on copepods, mainly driven by an increase in harpacticoid copepods, while a positive trend was also observed for polychaete larvae [31]. These results are consistent with previous studies demonstrating that warming can stimulate mesozooplankton development at the same location [37] and enhance zooplankton grazing in the Baltic sea [66]. Collectively, these findings highlight that the decrease in the abundances of protozooplankton and phytoplankton communities may be mediated by increased predation pressure exerted by mesozooplankton. “*

- Lines 525 – 532:

*“In addition, Soulié et al. (2022) reported that under MHW, the system tended to shift toward heterotrophy due to increased respiration and phytoplankton mortality [30], further supporting the idea that mesozooplankton exerted a strong top-down control on diatoms and protozooplankton under MHW condition. These outcomes could also impact biogeochemical cycles, particularly the oxygen cycle, where oxygen consumption may exceed production under MHW, with cascading consequences for the carbon cycle through an intensified release of carbon into the water. Consequently, under MHW, coastal Mediterranean systems may act as a carbon source to the atmosphere rather than as a sink of carbon.”*

These additions strengthen the discussion by clarifying how food web changes can affect biogeochemical process and overall ecosystem functioning.

**Reviewer #2 – comment 3:**

“Finally, conduct a thorough review to correct any grammatical or stylistic errors.”

**Reviewer #2 – response to comment 3:**

The entire manuscript was carefully revised to correct grammatical and stylistic errors. Additionally, we would like to point out that the entire manuscript has been reviewed by Editage, a recognized brand in the English edition (https://www.editage.com/) to ensure linguistic and grammatical accuracy prior to submission (an available certificate was issued by Editage).

We would like to thank the editor and reviewers once again for carefully evaluating our work. We believe that the revisions address the concerns raised and strengthen the manuscript. We look forward to your further consideration.

Sincerely,

Zoé Eglaine
