# Supplementary material for: Effects of a simulated marine heatwave on the structure and composition of Mediterranean plankton in a mesocosm study
Source: PLoS One. 2025 Nov 21;20(11):e0337112. doi: 10.1371/journal.pone.0337112 (PMC12637984; doi:10.1371/journal.pone.0337112)
Supplement: Attachment — Submitted filename: Response to Reviewers.docx [file pone.0337112.s008.docx]

Manuscript ID: **PONE-D-25-36140R1**

Title: **Effects of a simulated marine heatwave on the structure and composition of Mediterranean plankton in a mesocosm study.**

Dear Editor, Dear Reviewers,

We sincerely thank you again for the time and efforts that you dedicated to reviewing our manuscript and for your valuable and constructive comments. We have carefully considered all your remarks and revised the article accordingly. Below, we provide a detailed, point by point response to each of the reviewers’ comments.

We also describe the layout modifications made to clearly highlight the changes introduced in the second revised version of the manuscript in yellow (example: Adding text), while parts or elements removed from the manuscript are crossed out and colored red (example: ~~text deletion~~).

The numbers of lines mentioned below correspond to those of the “Second Revised Version Without Track Changes”.

**Journal Requirements:**

**Requirement 1:**

If the reviewer comments include a recommendation to cite specific previously published works, please review and evaluate these publications to determine whether they are relevant and should be cited. There is no requirement to cite these works unless the editor has indicated otherwise.

**Response 1:**

As no specific recommendation for additional citations was made by the Reviewers, no changes were made to the reference list.

**Requirement 2:**

Please review your reference list to ensure that it is complete and correct. If you have cited papers that have been retracted, please include the rationale for doing so in the manuscript text or remove these references and replace them with relevant current references. Any changes to the reference list should be mentioned in the rebuttal letter that accompanies your revised manuscript. If you need to cite a retracted article, indicate the article’s retracted status in the References list and also include a citation and full reference for the retraction notice.

**Response 2:**

We have carefully reviewed and updated the reference list to ensure its completeness and accuracy. All references were verified, and no retracted articles remain cited.

**Reviewers’ comments:**

**Reviewer #1 – comment 1:**

One additional point was noticed: line 219. Please rewrite it so that it does not imply that picoplankton cannot include cyanobacteria, for example. It usually consists of lots of cyanobacteria as well as other groups, but the way it is written implies that it is something completely different.
My suggestion would be: Resistance, resilience, recovery, and temporal stability were calculated according to the methods described by Hillebrand et al. [24] for viruses, bacteria, cyanobacteria, diatoms, dinoflagellates, flagellates for auto-, hetero- and mixotrophic, undetermined groups, HNF, and ciliates. Additionally, a distinct group of very small algal cells, referred to as picophytoplankton and nanophytoplankton were separated.

**Reviewer #1 – response to comment 1:**

We fully agree with this point and rewrite the sentence as suggested by Reviewer 1.

Lines 218 – 221:

*“Resistance, resilience, recovery, and temporal stability were calculated according to the methods described by Hillebrand et al. [24] for viruses, bacteria, cyanobacteria, diatoms, dinoflagellates, flagellates for auto-, hetero- and mixotrophic, undetermined groups, HNF, and ciliates. Additionally,* *a distinct group of very small algal cells, referred to as picophytoplankton and nanophytoplankton were separated”*

**Reviewer #1 – comment 2:**

On line 168, you wrote 'prokaryote' and 'eukaryote'. Please change these to 'prokaryotic' and 'eukaryotic'.

**Reviewer #1 – response to comment 2:**

We have replaced ‘prokaryote’ and ‘eukaryote’ by ‘prokaryotic’ and ‘eukaryotic’ in line 168.

Sincerely,

Zoé Eglaine and co-authors
